# Probiotic-derived ecto-5’-nucleotidase produces anti-inflammatory adenosine metabolites in Treg-deficient scurfy mice

**DOI:** 10.21203/rs.3.rs-2781715/v1

**Published:** 2023-04-07

**Authors:** Yuying Liu, Shabba A. Armbrister, Beanna Okeugo, Tingting W. Mills, Rhea C. Daniel, Jee-Hwan Oh, Jan-Peter Pijkeren, Evelyn S. Park, Zeina M. Saleh, Sharmistha Lahiri, Stefan Roos, J Marc Rhoads

**Affiliations:** the University of Texas Health Science Center at Houston; the University of Texas Health Science Center at Houston; the University of Texas Health Science Center at Houston; the University of Texas Health Science Center at Houston; the University of Texas Health Science Center at Houston; University of Wisconsin-Madison; University of Wisconsin-Madison; the University of Texas Health Science Center at Houston; the University of Texas Health Science Center at Houston; the University of Texas Health Science Center at Houston; Uppsala BioCenter, Swedish Univeristy of Agricultural Sciences; the University of Texas Health Science Center at Houston

**Keywords:** Limosilactobacillus reuteri, CD73, regulatory T cell, Treg-deficiency, autoimmunity, IPEX syndrome

## Abstract

Probiotic *Limosilactobacillus reuteri* DSM 17938 (DSM 17938) prolonges the survival of Treg-deficient scurfy (SF) mice and reduces multiorgan inflammation by a process requiring adenosine receptor 2A (A_2A_) on T cells. We hypothesized that *L. reuteri*-derived ecto-5’-nucleotidase (ecto-5’NT) activity acts to generate adenosine, which may be a central mediator for *L. reuteri* protection in SF mice. We evaluated DSM 17938–5’NT activity and the associated adenosine and inosine levels in plasma, gut and liver of SF mice. We examined orally fed DSM 17938, DSM 17938Δ5NT (with a deleted 5’NT gene), and DSM 32846 (BG-R46) (a naturally selected strain derived from DSM 17938). Results showed that DSM 17938 and BG-R46 produced adenosine while “exhausting” AMP, whereas DSM 17938Δ5NT did not generate adenosine in culture. Plasma 5’NT activity was increased by DSM 17938 or BG-R46, but not by DSM 17938Δ5NT in SF mice. BG-R46 increased both adenosine and inosine levels in the cecum of SF mice. DSM 17938 increased adenosine levels, whereas BG-R46 increased inosine levels in the liver. DSM 17938Δ5NT did not significantly change the levels of adenosine or inosine in the GI tract or the liver of SF mice. Although regulatory CD73^+^CD8^+^ T cells were decreased in spleen and blood of SF mice, these regulatory T cells could be increased by orally feeding DSM 17938 or BG-R46, but not DSM 17938Δ5NT. In conclusion, probiotic-5’NT may be a central mediator of DSM 17938 protection against autoimmunity. Optimal 5’NT activity from various probiotic strains could be beneficial in treating Treg-associated immune disorders in humans.

## Introduction

Regulatory T cells (Tregs) maintain immune homeostasis and play a pivotal role in immune tolerance [1]. Forkhead box protein 3 (Foxp3) is a major transcription factor that is associated with Treg cell development and function [2]. Genetic mutations or deletions of the Foxp3 gene result in a primary immunodeficiency (PID) disease known as IPEX (immunodysregulation, polyendocrinopathy, and enteropathy, with X-linked inheritance) syndrome in humans [3, 4]. The most common clinical presentation of IPEX syndrome is the classical triad of enteropathy, type I diabetes, and eczema, with early disease onset (the median age at disease onset 1.5–2 months) [3–7]. The scurfy (SF) mouse, bearing a mutation in the Foxp3 gene, displays a similar clinical phenotype with early onset dermatitis, progressive multiorgan inflammation, and death within the first month of life caused by a lymphoproliferative syndrome [8, 9].

One key mechanism by which Tregs control inflammatory effector T cells (Teffs) (including Th1 and Th2 cells) has been the adenosine pathway, wherein extracellular adenosine triphosphate (ATP) is promptly converted into adenosine through the coordinated activity of CD39 and CD73 (the latter the same as 5’-nucleotidase, 5’NT activity) [10]. CD39 and CD73 are highly expressed on the surface of Foxp3^+^Tregs and are increasingly used as markers of Tregs [11]. Upon Treg-derived adenosine’s release into the extracellular compartment, adenosine exerts anti-inflammatory functions via stimulation of specific G-protein-coupled receptors (adenosine receptors), which are predominantly expressed on Th1 and Th2 lymphocytes [12], inhibiting Th1/Th2 differentiation reducing inflammation [10]. In addition, adenosine generation triggers a self-reinforcing loop of Treg functions due to the stimulation of one adenosine receptor, A_2A,_ on Tregs, eliciting A_2A_ expansion, and enhanced Foxp3 expression, ans increasing immunoregulatory activity [10, 13].

The mechanism of Treg-derived adenosine to inhibit Th1/Th2 is unknown in human IPEX syndrome and SF mice. Previous studies found severe CD4^+^Th1 and Th2-cell-induced pathology in SF mice [14, 15] and in IPEX syndrome in humans [16, 17]. The intestinal microbiota drives host immune homeostasis by regulating the development T cells [18]. We have demonstrated the evolution of autoimmunity and microbial dysbiosis in the SF mice. We found that oral administration of probiotic *Limosilactobacillus reuteri* DSM 17938 (DSM 17938) remodeled gut microbiota and suppressed autoimmunity. DSM 17938 markedly prolonged the SF mouse’s lifespan from < 1 month to > 4 months [19].

Probiotics have the capacity to induce large-scale changes in the host microbiota composition and also to modulate the global metabolic function of the intestinal microbiome [20–22]. Probiotic DSM 17938 has been shown to have a mutualistic relationship between microbe and host [23–25]. Commercially available worldwide, DSM 17938 was derived from *Limosilactobacillus reuteri* ATCC 55730 (subcultured from a Peruvian mother’s breast milk) by removing two plasmids harboring antibiotic resistance genes [26]. DSM 17938 inhibits pathogen growth and modulates the immune system [21]. In humans, DSM 17938 also reduces the severity of acute infant diarrhea [27–29]; may reduce the incidence of necrotizing enterocolitis (NEC) in premature infants [30–33]; and has been shown to reduce crying time in babies with colic [34, 35].

We have tested the effects of DSM 17938 in several mouse models of human inflammatory and autoimmune diseases. We found this strain prevents experimental necrotizing enterocolitis (NEC) in newborn animals while inhibiting the toll-like receptor (TLR) 4-mediated NF-κB pathway [36], facilitating the induction of Tregs, and lowering the number of Teffs in the intestinal mucosa [37, 38] – by a mechanism requiring TLR2 [39]. In addition, DSM 17938 reduced the severity of experimental autoimmune encephalomyelitis (EAE), a mouse model of multiple sclerosis mainly driven by Th1- and Th17cell-induced inflammation [40]. Both of these conditions were associated with altered gut microbiota [41–43].

In testing the effect of DSM 17938 on SF mice, we identified a novel mechanism wherein the adenosine receptor 2A (A_2A_) expressed predominantly on T cells was required for probiotic protection, inasmuch as DSM 17938 had no therapeutic effect on SF mice with A_2A_ knocked out [19, 44]. We discovered that the adenosine metabolite *inosine* (formed from adenosine by the action of adenosine deaminase, ADA) was reduced in plasma of SF mice and was restored by DSM 17938 treatment, probably because DSM 17938 has ecto-5-nucleotidase (ecto-5’NT) activity comparable to CD73, producing adenosine. Oral administration of inosine to SF mice also prolonged the survival and controlled inflammation (in multiple organs) by acting as an A_2A_ agonist [19].

A major gap in probiotic biology has been our lack of understanding how DSM 17938 can activate the adenosine pathway and whether during Treg-deficiency, a probiotic-derived 5’NT activity, if present, could play an important role in generation of adenosine. In this study, we focused on evaluating the probiotic-derived 5’NT activity *in vitro* bacterial culture and the effect of probiotic-derived 5’NT on host plasma 5’NT activity, adenosine, and inosine levels.

## Material And Methods

### Mice

Wild-type (WT) C57BL/6J and heterozygous B6.Cg-Foxp3sf/J mice were purchased from Jackson Laboratories and allowed to acclimatize for 2 weeks before experimentation. SF mice with hemizygous B6.Cg-Foxp3sf/Y were generated from breeding heterozygous B6.Cg-Foxp3sf/J female (Jackson Lab 004088) to C57BL/6J male (Jackson Lab 000664). Only male mice were used in this study due to the Foxp3 gene existing on the X chromosome. In each litter of breeding pairs, all males were either SF used as experimental groups or WT littermates used as the control groups. all mice were housed in the animal facility at the UT Health Science Center at Houston. This study was carried out in accordance with the recommendation of *the Guide for the Care and Use of Laboratory Animals* (NIH) and the *Institutional Animal Care and Use Committee* (IACUC). The study was approved by The IACUC (protocol numbers: AWC-20–0032).

### Preparation of Probiotics

Probiotic DSM 17938 and DSM 32846 (BG-R46) were provided by BioGaia AB (Sweden). DSM 32846 (BG-R46) is a new probiotic strain obtained after selective breeding of DSM 17938 with higher levels of bile acid tolerance and adenosine production [45]. These probiotics were prepared as described previously [19]. Briefly, probiotics were anaerobically cultured in deMan-Rogosa-Sharpe (MRS) medium at 37°C for 24 h followed by plating in MRS agar at specific serial dilution and culturing anaerobically at 37°C for 48–72 h. quantitative analysis for bacteria in culture media was performed by comparing optical density (OD) at 600 nm of cultures at known concentrations using a standard curve of bacterial colony forming unit (CFU)/mL grown on MRS agar.

### Adenosine metabolism-associated enzymes: bacterial proteomic analysis

Sixteen-hour-DSM 17938 culture in MRS (n = 6) were collected and washed with phosphate buffered saline (PBS) three times before pellets were stored at −80° C until ready for use. Pellets were suspended in 500 μL of 40 mM Tris, 30 mM NaCl, pH 8.0, and protease inhibitor, then sonicated 6 times (10 s/cycle, 15 s in between cycles). All samples were then centrifuged at 15.4 *g* for 5 min at 10°C, and supernatants were saved. Protein concentrations of each samples were measured using DC^™^ Protein Assay (BioRad, Hercules, CA USA) according to manufactuer’s protocol. Aliquots of 50 μg of samples were sent to the Clinical and Translational Proteomics Service Center at the Institute of Molecular Medicine at The University of Texas Health Science Center at Houston for proteomic analysis, as described in our previous study [46]. The raw peptide data files were processed using Thermo Scientific Proteome discoverer TM software version 1.4 (Waltham, MA, USA). Spectra were searched against the *Limosilactobacillus reuteri* (previously *Lactobacillus reuteri*) strain ATCC 55730/SD2112, the parent strain of DSM 17938 [23] database using Sequest HT search engine [46].

By searching the genome of DSM 17938 [23] from UniProt (https://www.uniprot.org/) ELIXIR core data resources and confirmed proteins/genes in the National Center for Biotechnology Information (NCBI) (https://www.ncbi.nlm.nih.gov/protein/), we found that DSM 17938 contains a gene encoding a protein with homology to the 5’NT, named as LPXTG-motif cell wall anchor domain protein (https://www.uniprot.org/uniprot/F8DRN6) predicted to convert AMP to adenosine. Proteomics data have been reported in previous study [46]. We focused on detection of this enzyme (peptide) in DSM 17938 bacterial lysates, expressed as protein score (Score), the percentage of the protein sequence covered by identified peptides (Coverage), the number of distinct peptide sequences in the protein (#Peptides), and the total number of identified peptide sequences for the protein (#PSMs).

### Generation of 5’NT deletion strain of probiotic DSM 17938 (DSM17938Δ5NT)

To delete 5’NT gene in DSM 17938, we used a counter-selection plasmid pVPL3002 that is broadly applicable in lactobacilli [47]. Ligase cycling reaction (LCR) [48] was performed to clone the upstream (oVPL3468–3469) and downstream (oVPL3470–3471) flanks of 5NTE gene in pVPL3002 backbone (oVPL187–188) by using three bridging oligos (oVPL3472, 3473, and 3474) to yield pVPL31137 in *E. coli* EC1000 [47] (oligonucleotides/plasmid Table 1). First, pVPL31137 (2–5 μg) was electroporated in DSM 17938 followed by recovery in MRS (3 h at 37°C) and plating on MRS agar containing 5 μg/mL erythromycin. Erythromycin-resistant colonies were screened by colony PCR to confirm single-crossover homologous recombination using oVPL49-3475-3476 and oVPL97-3475-3476 for upstream and downstream integration of pVPL31137, respectively. Upon confirmation of single-crossover homologous recombination, a single colony was cultured in MRS for 20 h and plated on MRS agar containing 500 μg/mL vancomycin, which yields only colonies after a second homologous recombination event. Deletion of 5’NT gene was confirmed with oVPL3475–3476, and the sequence integrity was verified by Sanger sequencing. The resultant strain was named VPL4171 (DSM 17938Δ5NT).

### Probiotic culture preparation for measuring probiotic ecto-5’-nucleotidase (5’NT) activity

Overnight-cultured probiotics DSM 17938, BG-R46, and DSM17938Δ5NT were sampled to calculate CFU/mL, based on OD600nm against a standard curve. Immediately, 5 mL of bacterial cultures were treated with 400 μM AMP, 10 μM Deoxycoformycin (ADA inhibitor, from Sigma-Aldrich), and 10 μM Dipyridamole (nucleoside transport inhibitor, from Sigma-Aldrich); and 5 mL of control bacterial cultures had no added AMP. A “media control” had 5 mL of MRS bacterial culture medium. Cultures were incubated anaerobically at 37° C with shaking (220 rpm), and the culture supernatants were collected by centrifuge at 0 min, 5 min, 15 min, 30 min and 60 min, followed by storing at −80 °C for measuring adenosine and AMP levels by using reverse-phase high-performance liquid chromatography (HPLC). The adenosine levels were expressed as nmoles/10^9^ CFU bacteria. The AMP levels were expressed as μM.

### Treatment of mice and collection of blood and tissues for analysis

Newborn male mice (both SF and WT littermate) mice from SF breeding pairs stayed with their dams and were given by gavage DSM 17938, BG-R46 or DSM 17938Δ5NT in fresh MRS medium or control MRS medium. The experimental groups included WTC (WT male fed with MRS), SFC (SF mice fed with MRS), SF + DSM 17938 (SF mice fed with DSM 17938), SF + BG-R46 (SF mice fed with BG-R46), and SF + DSM17938Δ5NT (SF mice fed with DSM 17938Δ5NT). Oral gavage feeding started from day of life 10 (d10), once a SF phenotype could be recognized; gavage feeding was daily from d10 to d20, containing 10^7^ CFU in 100 μL. We used a Nutriline 1252.31G catheter (VYGON GmbH & Co., KG, Germany) to gavage the newborn mouse less than 12 d of age; and a polypropylene feeding tube (22-gauge 25 mm, Instech Laboratories, Inc., Plymouth Meeting, PA) to gavage the newborn mouse after d12 of age. At d21 (weaning date), blood and tissues including duodenum, jejunum, ileum, cecum, colon, liver, lung, and spleen were collected. To 200 μL of plasma, we immediately added nucleoside preservation cocktail (NPC) containing 10 μmole/L deoxycoformycin, 10 μmole/L dipyridamole, and 10 μmole/L-adenosine-5-a, b-methylene diphosphate (a CD73, 5’NT inhibitor, from Sigma-Aldrich) to prevent adenosine degradation. Samples were stored at −80°C freezer for further nucleoside purification and HPLC. Another 20 μL of plasma without addition of NPC was stored for measuring ecto-5’NT activity. Collected Tissues were freshly frozen and stored at −80°C for further processing to assess adenosine and inosine levels by HPLC.

### Plasma and tissue preparation for assessing the levels of adenosine, inosine, and AMP, activity 5’NT

Nucleosides were extracted from the plasma or the tissues using perchloric acid (PCA) extraction procedure [49]. Briefly, mouse plasma was collected or tissue lysates were prepared in the presence of protease inhibitor cocktail and nucleosides-preserving cocktail (10 μmole/L dipyridamole; 10 μmole/L deoxycoformycin, and 10 μmole/L αβ-methylene ADP), and plasma was mixed with 0.4 mole/L (0.4 N) PCA to precipitate the proteins. Samples were then neutralized with KHCO_3_/KOH, followed by acidification with NH_4_PO_4_ and phosphoric acid. The supernatant was collected after centrifugation and analyzed by reverse-phase HPLC as described previously [50–53]. Representative adenosine, inosine, and AMP peaks were identified and quantified using standard external curves. Data were normalized to the total protein amount of tissue lysates. 5’NT activities were measured as the amount of AMP converted to adenosine by each μL of plasma in 30 mins at 37 degrees. The samples were heat-inactivated at 95 degrees for 5 mins at the end of the reaction. Adenosine generated was measured using HPLC and presented as nmole adenosine/μL/min.

### Spleen single cell suspension preparation and blood cellsfor analyzing CD73 expression in T cells

Single cell suspensions from the spleen were obtained by gently fragmenting and filtering the tissues through 40-μm cell strainers (BD Bioscience, San Jose, CA) into RPMI 1640 (Sigma-Aldrich, St. Louis, MO) complete medium. MACS buffer consisting of phosphate-buffered saline, 0.5% bovine serum albumin (Hyclone GE Life Science, Logan, UT), and 2 mM EDTA (Lonza, Bethesda, MD) was used for washing the cells. Finally, cells were resuspended in MACS buffer for surface and intracellular staining using specific anti-mouse antibodies. The antibodies that we used were CD4 (GK1.5) conjugated with peridinin-chlorophyll protein/cyanine 5.5 (PerCP/Cy5.5), CD8a (53 − 6.7) conjugated with brilliant violet 421, CD39 (Duha59) conjugated with phycoerythrin (PE), CD73 (TY/11.8) conjugated with allophycocyanin (APC), and Foxp3 (150D) conjugated with Alexa Fluor 488 for T cell analysis. All antibodies were purchased from BioLegend (San Diego, CA). All samples were analyzed with BD LSRFortessa Flow Cytometer (BD Bioscience) and processed with FlowJo (FlowJo, BD, Ashland, OR).

### Statistical analysis

Significance was determined using one-way ANOVA for multiple comparisons with Tukey posttests, or two-way ANOVA for multiple comparisons with a Bonferroni test. Computed Pearson correlation coefficients analysis was used for correlation analysis. The statistical analysis was performed using GraphPad Prism version 9.4.1 (GraphPad Software, San Diego, CA). Data are represented as means ± SE. Values p < 0.05 were considered statistically significant.

## Results

### Gene encoded 5’NT peptides associated encoded adenosine with generation is detected by proteomics analysis

**Figure 1:**
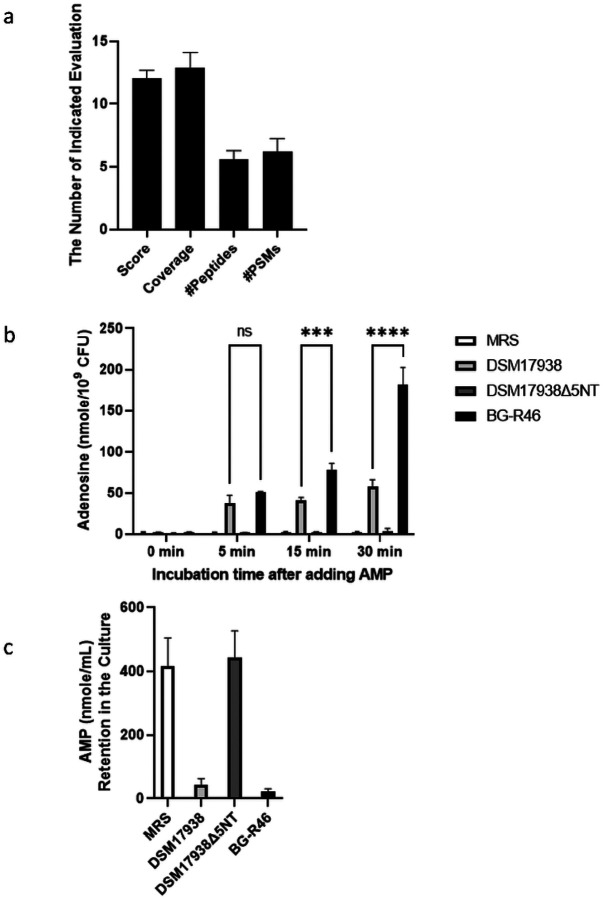
Probiotic-derived 5’NT activity in bacterial culture. a: Proteomics analysis detected 5’NT encoded peptides in bacterial lysates (n=6). b: Probiotic-derived 5’NT converting AMP to adenosine in culture medium with incubation time (n=4 indepednet experiments). c: AMP levels in the culture medium (n=4 independent experiments). ***p<0.001, ****p<0.0001.

Peptides that encoded by gene LPXTG-motif cell wall anchor domain protein (F8DRN6) with 5’NT function to convert AMP to adenosine were detected with different expression measurement units including protein score, coverage, number of peptides and number of PSMs (Fig. 1a) to confirm the transcript.

### DSM 17938 and BG-R46 have 5’NT enzymatic activity toconvert AMP to adenosine

We measured adenosine levels by HPLC in bacterial culture medium after addition of AMP. The adenosine levels were normalized by bacterial CFUs in culture to evaluate the ability of bacteria to convert AMP to adenosine by probiotic-5’NT; importantly, inhibitors were added to prevent adenosine degradation. The levels of AMP (nmole/mL = μM) in the culture medium were assessed by HPLC. We found that at as early as 5 min after addition of 400 μM of AMP to DSM 17938 bacterial culture, adenosine was detected, and the levels increased over the time of culture, whereas no adenosine was detected in DSM 17938Δ5NT cultures. The results also showed even higher levels of adenosine in cultures of probiotic BG-R46 (Fig. 1b). By measuring concentrations of AMP in the media, we showed that AMP was consumed and converted to adenosine by both strains, DSM 17938 and BG-R46.

### Probiotic-derived 5’NT plays a role in plasma levels of adenosine and inosine and 5’NT activity

**Figure 2:**
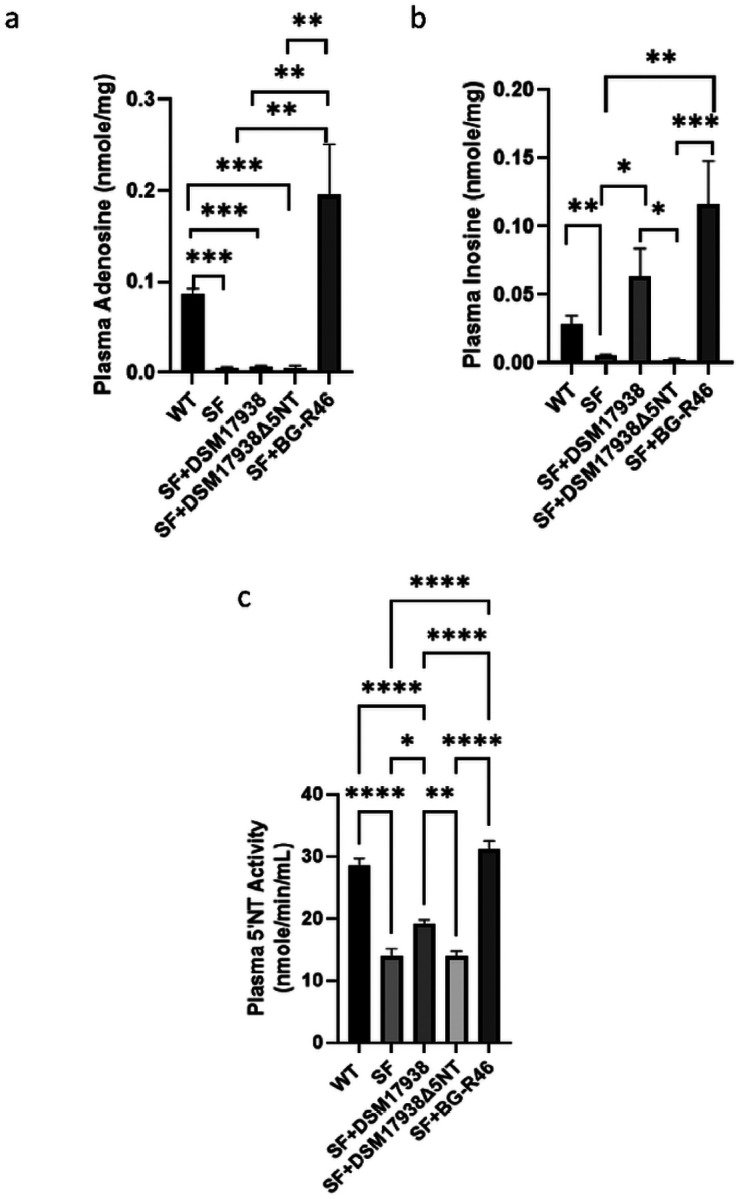
Plasma levels of adenosine, inosine and 5’NT activity in SF mice after feeding DSM 17938, BG-R46, or DSM 17938D5NT. a: plasma adenosine level in SF mice. b: plasma inosine level in SF mice. c: plasma 5’NT activity in SF mice. The levels were measured by HPLC. n=6–9 mice per group. *p<0.05, **p<0.01, ***p<0.001, and ****p<0.0001.

Plasma levels of adenosine and inosine at normal WT mice are very low: <0.1 nmole/mg total protein (for adenosine, Fig. 2a) and < 0.02 nmole/mg total protein (for inosine, Fig. 2b). SF mice showed significantly decreased plasma levels of adenosine and inosine compared to controls. However, oral gavage feeding of DSM 17938 significantly increased plasma inosine (Fig. 2b), while feeding BG-R46 to SF mice significantly increased plasma levels of adenosine and inosine. As expected, DSM 17938Δ5NT strain did not affect plasma levels of adenosine and inosine (Fig. 2a and 2b). Soluble 5’NT (CD73) activity in plasma was also measured; and results demonstrated that the 5’NT activity in SF mice was significantly reduced compared to WT mice, while 5’NT activity was increased by oral feeding of DSM 17938 or BG-R46. BG-46 was more potent than DSM 17938 in enhancing plasma 5’NT activity (Fig. 2c). However, DSM 17938Δ5NT did not affect 5’NT activity in SF mice, indicating DSM 17938-derived 5’NT plays an important role in adenosine metabolism in SF mice.

#### Probiotic-derived 5’NT increases levels of adenosine and inosine in the gut and liver of SF mice

**Figure 3:**
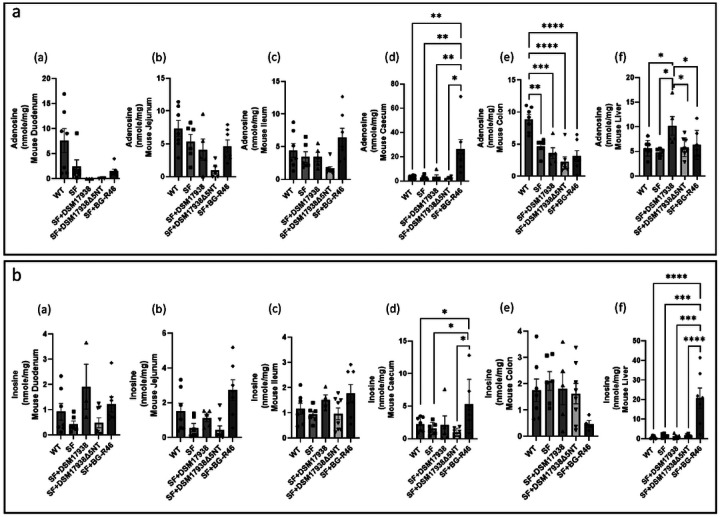
Adenosine and inosine levels in gut and liver of SF mice after probiotic treatment. a: Adenosine levels in duodenum (a), jejunum (b), ileum (c), cecum (d), colon (e), and liver (f). b: Inosine levels in duodenum (a), jejunum (b), ileum (c), cecum (d), colon (e), and liver (f). n=5–8 mice per group. Note that only in panels (d)-(f) were there any significant differences in regional expression. *p<0.05, **p<0.01, ***p<0.001, and ****p<0.0001.

We measured the levels of adenosine and inosine in different regions of the gastrointestinal tract (duodenum, jejunum, ileum, cecum, and colon) and in liver and spleen, and we normalized the levels from nmole/mL (by HPLC) to total protein concentration (mg/mL). Subsequently, the levels were expressed as nmole/mg total protein. We found that adenosine levels are < 10 nmole/mg; and no significant differences were observed between SF and WT mice; also, the adenosine levels were not significantly affected by probiotic treatment in the duodenum, jejunum, and ileum (Fig. 3a: (a)–(c)). However, we observed a significantly increased amount of adenosine in the cecum of SF mice when the mice orally feed with probiotic BG-R46 (Fig. 3-a: (d)). Note that, in the colon, the adenosine levels in SF mice were significantly reduced compared to that in WT mice, with no enhancement by probiotics (Fig. 3a: (e)). In the liver of SF mice, the adenosine levels were increased by DSM 17938 but not by BG-R46 and DSM 17938Δ5NT (Fig. 3a: (f)); whereas BG-R46 significantly increased levels of the metabolite inosine levels in the liver of SF mice (Fig. 3b: (f)).

Inosine could be measured in the duodenum, jejunum, ileum and colon; however, the concentrations were less than 5 nmole/mg and were not affected by probiotic treatment (Fig. 3b: (a)–(c) (e)) - except in the cecum, where BG-R46 significantly increased inosine levels (Fig. 3b: (d)), similarly to the increase in adenosine level in the cecum.

In summary, BG-R46 increased both adenosine and inosine levels in the cecum of SF mice. DSM 17938 increased adenosine levels in the liver of SF mice, whereas BG-R46 increased inosine levels in the liver of SF mice. Again, the 5’NT mutant strain DSM 17938Δ5NT did not change the levels of adenosine or inosine in the GI tract or the liver of SF mice (Fig. 3).

### Probiotic-derived 5’NT participates in immune regulation

**Figure 4:**
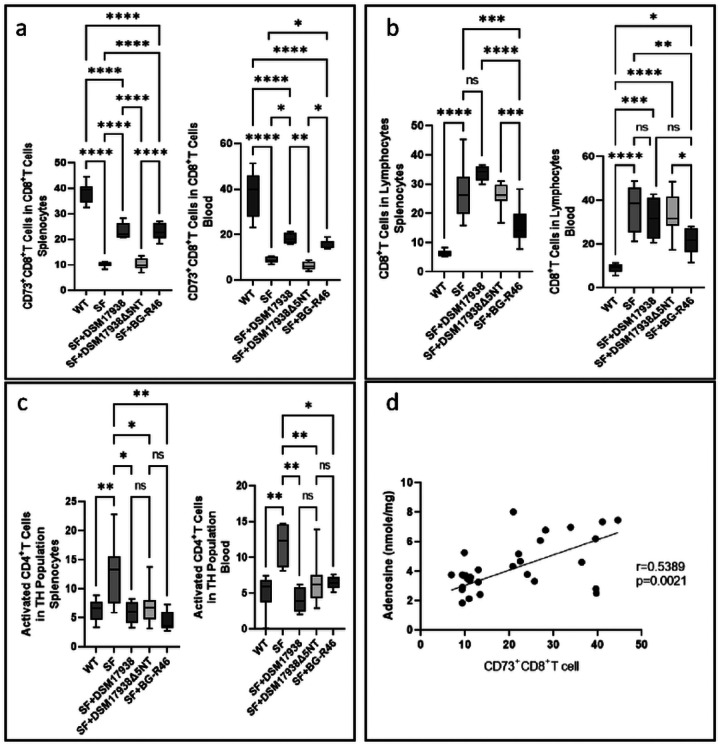
Regulatory CD73^+^CD8^+^T cells affected by orally feeding DSM 17938, BG-R46, or DSM 17938D5NT to SF mice. a: The proportion of CD73^+^CD8^+^T cells among CD8^+^T cells in spleen and blood analyzed by flow cytometry. b: The proportion of CD8^+^T cells in lymphocyte population in spleen and blood. c: The proportion of activated CD4^+^T cells among CD4^+^T cells in spleen and blood. d: The correlation between adenosine levels and CD73^+^CD8^+^T cells in the blood. n=6–9 mice per group. *p<0.05, **p<0.01, ***p<0.001, and ****p<0.0001.

Immune cells, specifically T cell populations in the spleen and blood, were analyzed by flow cytometry. We defined CD4^+^T cells and CD8^+^T cells in the lymphocyte population and further analyzed CD39 and CD73-expressing CD4^+^T cell and CD8^+^T cells and activated CD25^+^T cells. We found that CD73^+^CD8^+^ T cells were significantly decreased in both spleen and blood of SF mice, a lymphocyte populatoin which could be increased by orally feeding SF mice with either DSM 17938 or BG-R46. These probiotic-related increases did not reach the levels found in normal WT mice (Fig. 4a). Orally feeding of DSM 17938Δ5NT to SF mice did not change the percentage of CD73^+^CD8^+^ T cells (Fig. 4a), indicating probiotic 5’NT plays an important role in regulating this cell population. We also observed that SF mice had increased CD8^+^T cells in spleen and blood compared to that in WT mice, percentages which could be reduced by BG-R46, but not by DSM 17938 (Fig. 4b) (Fig. 4a).

SF mice are known to harbor increased activated CD4^+^T cells in the spleen and blood due to Foxp3^+^Treg deficiency. We observed that all 3 strains of lactobacilli (DSM 17938, BG-R46, and DSM 17938Δ5NT) were associated with reduced activated CD4^+^T cells in SF mice (Fig. 4c).

To test if probiotic-promoted adenosine levels are related to regulatory CD8^+^T cells, we performed a correlation analysis and found that adenosine levels in both spleen and blood were positively correlated with the percentage of CD73^+^CD8^+^ T cells (Fig. 4d).

## Discussion

Different probiotic strains have pleotropic effects. Some have bacteriocidal effects toward pathogens or pathobionts, others affect gut epithelial permeability, and still others are known to down-regulate inflammation. Our lab’s contribution has been to show that certain probiotics, such as *Limosilactobacillus reuteri* DSM 17938, may generate adenosine in the intestine, which is associated with reduced inflammation. This concept is based on our finding that the beneficial effect of DSM 17938 on a the scurfy mouse, a model of human IPEX syndrome, is dependent on adenosine receptor A_2A_ on T cells. The adenosine metabolite inosine was found to act as an agonist of its receptor to produce therapeutic effects [19, 44]. We found that Treg-derived adenosine interacted with A_2A_ expressed on Teff to inhibit Th cell differentiation and reduce inflammation. Formation of extracellular adenosine requires CD39 and CD73 present on Treg cells [54]. In Treg-deficient SF mice, this protective mechanism is disrupted.

Mechanistically, adenosine may be generated by modulation of host gut enzymatic activities and/or by microbial enzymes. DSM 17938 contains a gene encoding a secreted ecto 5’nucleotidase (5’NT). In probing a newly discovered mechanism of action of DSM 17938, we aimed to determine whether microbial 5’NT plays a role in adenosine production. In this study, we confirmed that DSM 17938 produced adenosine while “exhausting” AMP. DSM 17938Δ5NT had no effect on adenosine production.

Mammalian CD73 is a 71kDa homodimer which can be a membrane-bound phospholipase or a soluble form of the protein [55]. Host soluble CD73 can be purified from tissues such as human placenta and shows a similar affinity for AMP as the membrane-bound form [56]. Previous studies showed that the host soluble CD73 can result from protein shedding through hydrolysis by endogenous membrane phospholipase or by proteolytic cleavage [55, 57]. In our study, SF mice have lower levels of plasma 5’NT (CD73) activity compared to those of WT mice, indicating that there is likely reduced endogenous CD73 generated by host cells such as intestinal epithelial cells and/or immune cells in SF mice. We showed that both probiotic strains DSM 17938 and BG-R46 increased mouse plasma 5’NT enzymatic activity in SF mice, whereas the mutant strain with a deleted 5’NT gene was inactive (with no change in the mouse plasma level of 5’NT activity) in SF mice. We inferred that this increased soluble 5’NT activity in peripheral blood of SF mice most likely originated from the probiotic. It is possible that probiotic DSM 17938-derived 5’NT in the intestine caused from microbial shedding to yield the free form to the circulating blood; but it is also possible that bacterial 5’NT in the intestine upregulated endogenous CD73 from enterocytes and/or from lamina propria immunue cells that was released as the free form of 5’NT. We showed in this study that bacterial 5’NT did promote endogenous CD73 expression on T cells.

CD73 is one of a number of ectoenzymes that are present in soluble form in peripheral blood. Other enzymes that participate in purine-metabolism, such as alkaline phosphatase, adenosine deaminase, ATP-degrading enzyme (CD39) and ATP-regenerating kinases are also present, indicating a complex network of enzymatically active molecules that can counterbalance purinergic signaling to modulate the immune response [55]. Specifically, one of the functions of this network is involved in maintaining Treg cell stability [58]. In Foxp3^+^Treg deficient SF mice, it appears that the balance of purine metabolism is disrupted, because our results show reduced levels adenosine, inosine, and CD73 activity in plasma, along with significantly reduced populations of CD73^+^CD8^+^T cells in spleen and blood.

Studies in healthy infants demonstrate that probiotics cannot permanently change intestinal microbiota community structure or diversity [59], and colonization resistance may be one of the important reasons for limitation of the long-term effects of probiotics. The SF mouse has been characterized by gut microbial dysbiosis [19]. Our finding in this study that BG-R46, a naturally selected strain from DSM 17938 [45], significantly increased adenosine levels in cecum in the SF mice after gavage feeding. We hypothesize that the cecum may be a site for generating adenosine and its metabolite inosine, further being absorped and translocated to sites such as the liver to execute its anti-inflammatory actions. In fact, we found that BG-R46 increased levels of adenosine in the plasma, while both probiotics increased levels of inosine in plasma and liver. It should be noted that studies in mouse models and human cells and tissues have identified that the production of adenosine and its subsequent signaling through its receptors plays largely beneficial roles in acute or early disease states such as inflammation [60]. In contrast, beyond the acute/early phase, adenosine signaling can become detrimental and promote tissue injury and fibrosis such as lung or liver fibrosis [61, 62]. Probiotic-derived adenosine and its metabolite inosine have beneficial effects in SF mice when adenosine-producing DSM 17938 is given before or shortly after SF mice exhibit significant multi-organ inflammation in the liver and lung [19, 44].

*In vivo*, the converstion of adenosine to inosine may happen rapidly because of the much shorter biological half-life of adenosine compared with inosine [63]. The observation of sometimes discrepant adenosine and inosine levels (for example, increased adenosine by DSM 17938 versus increased inosine by BG-R46 in the liver of SF mice) could be an artifact of rapid conversion and perhaps less than 100% blocking of ADA by deoxycoformycin. In our previous studies, we showed that DSM 17938 feeding increases in the levels of adenosine or inosine expressed either as a relative fold change or an intensity scale in the plasma of healthy newborn [64] or Treg-deficienct SF mouse [19, 65]. The current study provided absolute values of measured adenosine and inosine and confirmed the observations from our previous studies.

Recent studies have shown that bacterial extracellular membrane vesicles isolated from BG-R46 and DSM 17938 have 5’NT activity *in vitro*, and their administration upregulated IL-1beta and IL-6 produced by peripheral blood mononuclear cells (PBMCs) but dampened IFN-γ and TNF-α responses in PBMC challenged with *Staphylococcus aureus* [45]. In Treg-deficienct SF mice, possible mechanisms for high levels of adenosine and inosine in the plasma and liver after probiotic feeding include: (a) probiotic-5’NT generated from adenosine/inosine in the cecum may be transported via nucleotidase transporters to the blood; (b) soluble or MV bound probiotic-derived 5’NT could be released into the blood and could have enzymatic activity in the liver to convert AMP to adenosine; and (c) higher levels of ATP in the inflamed liver in SF mice could provide a source for probiotic-derived 5-NT to generate more adenosine or inosine.

CD73^+^CD8^+^T cells have been shown to have regulatory functions relevant to autoimmune disorders. Recent studies showed CD8^+^T cells yielded extracelluar vesicles (EVs) into cell culture supernatants with enzymatically active CD73. These activated CD73^+^CD8^+^T cell-derived EVs are immunosuppressive, for example in patients with juvenile idiopathic arthritis [66]. In SF mice, the % of CD73^+^CD8^+^T cells significantly decreased in the spleen and blood compared to that in WT mice. We hypothesized that DSM 17938 and BG-R46-derived 5’NT activity could enhance the proportion of CD73 expressing CD8^+^T cells in spleen. We found that 5’NT-mediated adenosine generation positively correlated with CD73^+^CD8^+^T cell numbers.

The inflammation of SF mice due to Treg-deficiency is mainly associated with Th1 and Th2 responses [14, 15, 19]. In the current study, we found an increase in activated CD4^+^T cells in the spleen and blood of SF mice compared to WT mice, while probiotic DSM 17938 and BG-R46 reduced the percentage of activated CD4^+^T cells. Interestingly, DSM 17938Δ5NTE also reduced activated CD4^+^T cells, indicating other possible anti-inflammatory mechanisms of this probiotic. As an example of another anti-inflammatory mechanism, we previously found in a NEC model that DSM 17938 promotes tolerogenic DCs via TLR2 to reduce inflammation [39]. The membrane vesicles isolated from BG-R46 and DSM 17938 carry the TLR2 agonist lipoteichic acid [45].

In conclusion, probiotic-derived 5’NT plays a central role in DSM 17938-mediated protection against autoimmunity in Treg-deficient SF mice. In humans, primary immunodeficiency diseases associated with Treg-deficiency/dysfunction are not limited to IPEX syndrome. There are a number of IPEX-like syndromes with a normal FoxP3 genotype, as well as other single gene defects that affect the function of Tregs, including CD25, the signal transducer and activator of transcription (STAT)5b, itchy E3 ubiquitin protein ligase (ITCH), the cytotoxic T lymphocyte antigen-4 (CTLA4), and Wiskott-Aldrich syndrome gene (WAS) [67]. Mechanistic insights in adenosine generation by probiotic-5’NT that are involved in immune regulation could eventually allow screening of various probiotic strains to determine which is capable of nucleoside signaling to be used for treating Treg-associated immune disorders in humans.

## Figures and Tables

**Table 1:** Primers used for amplifying u/s and d/s flanking sequence

SCO screening oligo (d/s)	oVPL49	acaatttcacacaggaaacagc
SCO screening oligo, (u/s)	oVPL97	cccccattaagtgccgagtgc
u/s flank Fwd	oVPL3468	tgctgatcaggcgtatggta
u/s flank Rev	oVPL3469	tatagtcctaacagcgctgtcc
d/s flank Fwd	oVPL3470	agctgaccagcaagcagctt
d/s flank Rev	oVPL3471	ctgaccatgatggagagcaa
Bridging oligo1	oVPL3472	aaacgacggccagtgaattcgagctcggtatgctgatcaggcgtatggtagatttttgaa
Bridging oligo2	oVPL3473	attgttagggacagcgctgttaggactataagctgaccagcaagcagctttaccacaaac
Bridging oligo3	oVPL3474	atccagttaattgctctccatcatggtcagatcctctagagtcgacctgcaggcatgcaa
DCO screening oligo, Fwd	oVPL3475	cagtcaaatcggccttcatt
DCO screening oligo, Rev	oVPL3476	ttactcgcatttggtgagca

d/s: downstream, u/s: upstream, SCO: single-crossover, DCO: double-crossover

## Data Availability

Most data generated or analysed during this study are included in this published article. Those data generated and/or analysed during the current study that are not published here are available from corresponding author on reasonable request.
